# Validation of general pain scores from multidomain assessment tools in stroke

**DOI:** 10.3389/fneur.2024.1328832

**Published:** 2024-01-25

**Authors:** Myzoon Ali, Holly Tibble, Marian C. Brady, Terence J. Quinn, Katharina S. Sunnerhagen, Narayanaswamy Venketasubramanian, Ashfaq Shuaib, Anand Pandyan, Gillian Mead

**Affiliations:** ^1^School of Cardiovascular and Metabolic Health, University of Glasgow, Glasgow, United Kingdom; ^2^NMAHP Research Unit, Glasgow Caledonian University, Glasgow, United Kingdom; ^3^Centre for Medical Informatics, Usher Institute, University of Edinburgh, Edinburgh, United Kingdom; ^4^Department of Clinical Neuroscience, University of Gothenburg, Sweden and Sahlgrenska University Hospital, Gothenburg, Sweden; ^5^Raffles Neuroscience Centre, Raffles Hospital, Singapore, Singapore; ^6^Division of Neurology, Department of Medicine, University of Alberta, Edmonton, AB, Canada; ^7^Faculty of Health and Social Sciences, Bournemouth University, Poole, United Kingdom; ^8^Geriatric Medicine, Division of Health Sciences, University of Edinburgh, Edinburgh, United Kingdom

**Keywords:** stroke, pain, assessment optimisation, EQ-5D-3L, EQ-5D-5L, NPRS stroke, assessment, EQ-5D

## Abstract

**Purpose:**

We describe how well general pain reported in multidomain assessment tools correlated with pain-specific assessment tools; associations between general pain, activities of daily living and independence after stroke.

**Materials and methods:**

Analyses of individual participant data (IPD) from the Virtual International Stroke Trials Archive (VISTA) described correlation coefficients examining (i) direct comparisons of assessments from pain-specific and multidomain assessment tools that included pain, (ii) indirect comparisons of pain assessments with the Barthel Index (BI) and modified Rankin Scale (mRS), and (iii) whether pain identification could be enhanced by accounting for reported usual activities, self-care, mobility and anxiety/depression; factors associated with pain.

**Results:**

European Quality of Life 3- and 5-Level (EQ-5D-3L and EQ-5D-5L), RAND 36 Item Health Survey 1.0 (SF-36) or the 0–10 Numeric Pain Rating Scale (NPRS) were available from 10/94 studies (IPD = 10,002). The 0–10 NPRS was the only available pain-specific assessment tool and was a reference for comparison with other tools. Pearson correlation coefficients between the 0–10 NPRS and (A) the EQ-5D-3L and (B) EQ5D-5 L were *r* = 0.572 (*n* = 436) and *r* = 0.305 (*n* = 1,134), respectively. mRS was better aligned with pain by EQ-5D-3L (*n* = 8,966; *r* = 0.340) than by SF-36 (*n* = 623; *r* = 0.318). BI aligned better with pain by SF-36 (*n* = 623; *r* = −0.320). Creating a composite score using the EQ-5D 3 L and 5 L comprising pain, mobility, usual-activities, self-care and anxiety/depression did not improve correlation with the 0–10 NPRS.

**Discussion:**

The EQ-5D-3L pain domain aligned better than the EQ-5D-5L with the 0–10 NPRS and may inform general pain description where resources and assessment burden hinder use of additional, pain-specific assessments.

## Introduction

Post-stroke pain is common (1, 2), varies in aetiology, can affect up to 70% (3–6) of people, has a 1-year prevalence of between 11% (7) and 48% (8) and is associated with poorer quality of life (QoL) (9). Pain and physical inactivity are inter-related. Low levels of physical activity have been linked with presence of post-stroke pain (10); increased physical activity can reduce the risk of chronic pain (11) and alleviate pain symptoms (12). The presence of pain can impact on a person’s ability to participate in rehabilitation (13), further compounding mobility issues, and thereby increasing the presence of pain (14).

Pain experienced after stroke can include central post-stroke pain (CPSP), musculoskeletal pain, complex regional pain syndrome, pain due to spasticity, hemiplegic shoulder and pre-existing conditions such as arthritis (15). Assessment of pain is necessary to inform management and intervention. Despite its prevalence, impact on mobility and engagement with rehabilitation, assessment and management of pain are often neglected (16). When included in stroke research studies, pain assessments typically comprise patient questionnaires, use self-reported scales or are embedded within multidomain assessments of general health (17). A systematic review of pain assessment in stroke identified 10 pain tools from 12 stroke studies (1), including the Visual Analogue Scale (VAS) for pain, the Faces Pain Scale (FPS), the 0–10 Numeric Pain Rating Scale (NPRS), the Pain Assessment Scale for Seniors with Severe Dementia-II, the AbilityQ, ShoulderQ, and the Neuropathic Pain Diagnostic Questionnaire (1). Of these, the most commonly used scale was the FPS.

Selection of appropriate pain assessment tools for use in clinical research needs to balance scale reliability, validity, availability in clinical contexts and across languages, and test-burden (1). Compared with age-matched controls, and excluding people who have language impairment (aphasia) or reduced levels of consciousness, people with stroke are less likely to be able to complete certain clinical rating scales (18). Self-reporting of pain also underestimates the extent of pain. Almost 40% of stroke survivors who did not declare presence of shoulder pain when asked, demonstrated pain upon physical examination (19). Given the range and prevalence of consequences such as communication (20) and cognitive impairments (21), no single pain assessment scale appears to be administrable for all people with stroke (22); this adds complexity to the selection of appropriate post-stroke pain assessment tools, identification and management of pain after stroke.

Despite its importance, we lack consensus on the optimum measure to assess pain in this population (1, 2). Multidomain assessment tools may offer capture of a range of outcomes and can be less burdensome than administration of multiple assessment tools to address each domain of interest. We examined how well pain that is captured in multidomain assessment tools correlates with assessment tools designed to quantify pain intensity (pain-specific measures).

## Materials and methods

We conducted retrospective analyses of pooled clinical trial data from the Virtual International Stroke Trials Archives (VISTA).

### Ethical approval

Analysis of data from the Virtual Trials Archives: VISTA, VICCTA and VIRTTA have been approved by the University of Glasgow’s MVLS College Ethics Committee (Project number 200170016).

### Inclusion criteria

Our eligibility criterion was assessment of pain. We extracted Individual Participant Data (IPD) on age, sex, medical history variables, time since stroke onset, available pain assessments, mobility using the Barthel Index (BI), independence using the modified Rankin scale (mRS) scores, presence of a language impairment [≥1 on the Best Language domain of the National Institutes of Health Stroke Scale (NIHSS) at baseline] and index stroke severity. We identified pain-specific assessment tools, and pain items from multidomain assessment tools. Throughout, if multiple assessments of pain were available for the same scale within the same time-period within an individual, the median value was calculated.

### Direct comparisons of pain assessment tools

Each pain scale described pain severity. We calculated the Pearson correlation coefficient to examine the strength and direction of the linear relationship between pain-specific scales and those that were from multidomain assessment tools, assessed within the same time window, where data were available. The Spearman correlation coefficient was also calculated as a sensitivity analysis of the linearity of the relationship between the variables.

### Indirect comparisons of pain assessment tools

We examined the Pearson correlation coefficient and Spearman correlation between each pain assessment tool and measurements of everyday activity (BI) and independence (mRS).

### Creation of a composite score to enhance the capture of pain

Acknowledging the relationship between mobility (10), activities of daily living (ADLs) (23) and depression (14), we examined methods to optimise the capture of pain in multidomain assessment tools. We investigated whether adjustment for scores in the European Quality of Life Scale (EQ-5D) 3 and 5 Level domains of anxiety, mobility, self-care, and usual activities enhanced the capture of pain, by examining multiple correlation coefficients with pain-specific scales.

We created a composite score including the EQ-5D domains of mobility, self-care, usual activities and anxiety/depression along with pain. We described whether this composite score enhanced the capture of pain by examining alignment with pain-specific assessment tools, where available.

## Results

### Study population

Of 94 studies in VISTA (> 48,000 participants), we identified 10,002 individuals from nine RCTs and an additional RCT with an embedded cohort study, for whom pain was assessed after stroke onset. Median age was 70 years (interquartile range 59.0–77.1 years), 55.6% were male (*n* = 5,560), and 37.4% had aphasia at baseline (*n* = 2,269/6,066; Table 1). Recruitment took place in acute (enrolment <24 h, *n* = 4,877), non-acute (enrolment >1 month, *N* = 1,102) and mixed settings (within 7 days to 1 month, *n* = 4,023). Post-stroke pain assessments were available for 132 (1.3%) people <1 month post-stroke, 5,094 (50.9%) from 1 to 3 months, 865 (8.6%) from 4 to 6 months, and 4,776 (47.8%) from 6 to 12 months post-stroke.

**Table 1:** Baseline characteristics.

Variable		IPD (*N* = 10,002)
Age (median [IQR] years)		70.0 (59.0–77.1)
Sex (*n* male; %)		5,560 (55.6%)
Time from stroke onset to recruitment; days (median [IQR])		1.4 (1–7)
Stroke type		
	Confirmed Ischaemic	5,421 (54.2%)
	Presumed Ischaemic	30 (0.3%)
	Intracerebral Haemorrhage (ICH)	625 (6.2%)
	Mixed (ischaemic & ICH)	4 (<0.1%)
	Subarachnoid Haemorrhage (SAH)	10 (0.1%)
Baseline National Institutes of Health Stroke Scale Score (NIHSS; median [IQR])		10 (7–15)
Aphasia at baseline (*n* yes; %)		2,269 (37.4%)

Self-reported pain was available for 5,167/10,834 (48%) participants using the EQ-5D-3L. The median self-reported pain score on the EQ-5D-3L was 1 (IQR [1,2]), while the median proxy-reported pain score was 2 (IQR [1,2]; Wilcoxon Rank Sum *p* < 0.01).

Only one pain-specific measure was available from two studies (0–10 Numeric Pain Rating Scale; NPRS; *n* = 1,100), while three multidomain assessment tools included assessment of pain (EQ-5D-3L; EQ-5D-5L and the RAND 36 Item Health Survey 1.0 item 21; SF-36). Pain was commonly assessed using the EQ-5D-3L (8/10 studies); one study used the EQ-5D-5L and one study used the SF-36 (24).

### Direct comparisons of pain assessment tools

Only two studies within our dataset used more than one pain scale, allowing investigation of the correlation between pain-specific measures and the pain items from multidomain assessment measures. The Pearson correlation coefficient between the EQ-5D-3L and the 0–10 NPRS (matched by timepoint) was 0.572 [*p* < 0.001, *n* = 436, *R*(2) = 0.327]. There was no substantial difference between the Pearson and Spearman correlation coefficients (rho = 0.575) or between the *R*^2^ before and after adjusting for age and sex (*R*^2^ = 0.328).

The Pearson correlation coefficient between the EQ-5D-5L and the 0–10 NPRS was 0.305 (*p* < 0.001, *n* = 1,134, *R*^2^ = 0.093). There was little difference when the Spearman correlation coefficient was used (rho = 0.311) or after adjusting for age and sex (*R*^2^ = 0.094).

### Indirect comparisons of pain assessment tools through individual association with ADLs and independence

No studies captured data on both the mRS and pain (by either EQ5D-5 L or the 0–10 NPRS). Therefore, indirect comparisons were conducted between mRS with SF-36 and EQ-5D-3L, and BI with the SF-36, EQ-5D-3L, EQ-5D-5L and the 0–10 NPRS.

The SF-36 had the strongest (moderate) Pearson correlation with the BI (Table 2) in unadjusted analyses (*r* = −0.320) and the *R*^2^ was 0.102 when adjusted for age and sex. The EQ-5D-3L had the strongest correlation with the mRS (*r* = 0.340, *R*^2^ = 0.117).

**Table 2:** Associations between pain scales and everyday function.

Pain scale	Everyday function scale	Sample size	Pearson correlation coefficient			Age- and sex-adjusted coefficient of determination	Spearman correlation coefficient	
			*r*	*p* value	*r* ^2^		rho	*p* value
						*R* ^2^		
SF36	Modified Rankin Scale (mRS)	623	0.318	<0.001	0.101	0.102	0.309	<0.001
EQ-5D-3L		8,966	0.340	<0.001	0.116	0.117	0.337	<0.001
SF36	Barthel index	623	−0.320	<0.001	0.102	0.102	−0.340	<0.001
EQ-5D-3L		5,499	−0.280	<0.001	0.078	0.081	−0.316	<0.001
EQ-5D-5L		1,135	−0.018	0.545	<0.001	0.001	−0.037	0.217
0–10 NPRS		1,594	−0.097	<0.001	0.009	0.013	−0.115	<0.001

### Adjustment of scores to enhance the capture of pain

After adjusting for age, sex, and EQ-5D-3L scores for mobility, usual activities, self-care, and anxiety, the multiple correlation coefficient between the 0–10 NPRS and EQ-5D-3L pain scores increased from *R* = 0.572 (unadjusted) to *R* = 0.600 (5% improvement; Table 3). After adjusting for age, sex, and EQ-5D-5L scores for mobility, usual activities, self-care and anxiety, the *R*^2^ between the 0–10 NPRS and EQ-5D-5L pain scores increased from 0.305 (unadjusted) to 0.439 (44% improvement; Table 4).

**Table 3:** Adjustment and composition of scores from the EQ-5D-3L, compared to pain on the 0–10 numeric pain scale.

EQ-5D-3L pain	Mobility	Anxiety and/or depression score	Usual activities	Self-care	Multiple correlation coefficient from adjusted model (*R*)	Pearson correlation coefficient with composite measure (*r*)
+	−	−	−	−	0.572	
+	+	−	−	−	0.582	0.581
+	−	+	−	−	0.588	0.586
+	−	−	+	−	0.574	0.570
+	−	−	−	+	0.578	0.578
+	+	+	−	−	0.597	0.594
+	+	−	+	−	0.583	0.575
+	+	−	−	+	0.584	0.581
+	−	+	+	−	0.588	0.583
+	−	+	−	+	0.592	0.590
+	−	−	+		0.578	0.570
+	−	+	+	+	0.593	0.581
+	+	−	+	+	0.584	0.571
+	+	+	−	+	0.598	0.594
+	+	+	+	−	0.598	0.588
+	+	+	+	+	0.600	0.583

+ indicates the inclusion of the EQ-5D-3L domain in the adjusted model or the composite score.

**Table 4:** Adjustment and composition of scores from the EQ-5D-5L, compared to pain on the 0–10 numeric pain scale.

EQ-5D-5L pain	Mobility	Anxiety and/or depression score	Usual activities	Self-care	Multiple correlation coefficient from adjusted model (*R*)	Pearson correlation coefficient with composite measure (*r*)
+	−	−	−	−	0.305	
+	+	−	−	−	0.370	0.357
+	−	+	−	−	0.390	0.362
+	−	−	+	−	0.369	0.357
+	−	−	−	+	0.377	0.362
+	+	+	−	−	0.421	0.401
+	+	−	+	−	0.389	0.386
+	+	−	−	+	0.391	0.388
+	−	+	+	−	0.423	0.403
+	−	+	−	+	0.426	0.406
+	−	−	+		0.390	0.388
+	−	+	+	+	0.435	0.423
+	+	−	+	+	0.399	0.399
+	+	+	−	+	0.434	0.423
+	+	+	+	−	0.434	0.422
+	+	+	+	+	0.439	0.428

+ indicates the inclusion of the EQ-5D-5L domain in the adjusted model or the composite score.

### Creation of composite scores to enhance the capture of pain

The two composites of EQ-5D-3L domains with the highest Pearson correlation coefficient with the 0–10 NPRS were Pain + Mobility + Anxiety/Depression (score range 3–9) and Pain + Mobility + Anxiety/Depression + Self Care (score range 4–12), both with *r* = 0.594 (4% increase from pain score alone; Table 3). For EQ-5D-5L domains, the best composite was Pain + Mobility + Anxiety/Depression + Usual Activities + Self Care (score range 5–15), with *r* = 0.428 (40% increase; Table 4). The composite scores therefore did not improve the capture of pain compared to adjusted analyses.

## Discussion

The EQ-5D-3L was the most commonly used multidomain scale that included pain assessment; this had a moderate *R*^2^ of 0.327 with the 0–10 NPRS. Surprisingly, the five-level EQ-5D did not capture pain as well as the three-level scale, when compared to the 0–10 NPRS (*R*^2^ = 0.093). We were unable to examine the correlation between the mRS and 0–10 NPRS in our dataset due to unavailability of corresponding datapoints, but previous work reported a weak relationship between pain and the mRS (25–27). We observed a correlation of −0.097 between the BI and the 0–10 NPRS; considerably weaker than previous work describing associations between the 0–10 NPRS and the Korean Instrumental Activities of Daily Living (K-IADL), reporting a correlation of 0.374 (28). The capture of pain by EQ-5D-3L was not improved by considering participants’ ability to care for themselves, complete ADLs, presence of anxiety/depression or overall health state. Consequently, the pain domain of the EQ-5D-3L on its own appeared to be a somewhat adequate marker for the presence of pain.

As the 0–10 NPRS was the only pain-specific assessment tool from 94 studies in VISTA, we compared this with the pain items from multidomain assessment tools. The 0–10 NPRS is a simple, practical and understandable pain assessment tool (29); with short administration time (30) and is suitable for use over the telephone (31), and in people with visual acuity or dexterity problems (32), both of which are often seen in stroke populations. The 0–10 NPRS aligns well with other pain measurement tools (33, 34). However, despite its previous use in stroke populations (35, 36), work is still needed to validate its use in this population. Additionally, point increases on this scale are not proportionate to pain experienced (37). Nevertheless, in the absence of other pain-specific measures that have been validated for use in the stroke population, it was deemed to be a suitable pain-specific assessment tool to which pain in multidomain assessment tools could be compared in our study.

Previous work has reported good reliability of the NPRS (38); the Functional Pain Scale correlated strongly with the NPRS, though mean scores between both differed significantly (37). Additionally, the NPRS showed high test–retest ability and is highly correlated with the pain Visual Analogue Scale (VAS) (39). Docherty et al. (40) reported moderate correlation between the EQ-5D-5L pain and discomfort domain with the Brief Pain Inventory (BPI). A separate study in adult burn patients reported Spearman correlation of 0.468 between the EQ-5D-5L pain and discomfort domain and the Patient and Observer Scar Assessment Scale (POSAS) (41). These results are congruent with our findings and further lend evidence to the moderate capture of pain within the EQ-5D pain domain.

Our study has some limitations. Our sample comprised selected studies that were represented in this international archive and was not based on a systematic identification of eligible dataset from the literature; thus further RCT data with measures of pain exist but were not represented in this analysis. Our dataset of 10,002 IPD were drawn from 10 studies, where presence or intensity of pain were not the primary endpoint, but where pain had been captured as either a secondary outcome or as an item in a multidomain assessment. Therefore, the range of assessment tools available from VISTA did not include some of the most common post-stroke pain assessments previously identified by Edwards et al. (1). Additionally, we were only able to compare pain in multidomain assessment tools to a single pain-specific assessment tool. There were no data available on participants who were assessed using both the SF-36 and the 0–10 NPRS. We therefore could not quantify the association between these pain scales to compare with the strength of association between the EQ-5D 3 and 5 L, and the 0–10 NPRS. Similarly, lack of overlapping data within participants meant that we could not fully examine correlations between pain assessments and independence by mRS.

The EQ-5D-3L, 5 L and NPRS each assess pain intensity, and do not include impact of pain on daily life, nor specify the location or aetiology of pain. While the SF-36 included a domain to assess how pain interfered with normal life, we described pain using the domain that captured pain intensity in our analyses. Presence of pain could typically be captured as a binary outcome (present/absent); as each measurement tool in our sample captured pain intensity, we pragmatically described presence of pain as any score above the scale minimum (which corresponded to no pain on all assessment tools).

Pain as a result to damage to neural structures can be associated with damage to the thalamus or other ascending sensory pathway structures (42). We were unable to account for anatomical differences among strokes in our sample. Similarly, we were not able to account for history of arthritis, which would be expected in an ageing population. Future research would benefit from prospective collection of data relevant to the post-stroke pain population including history of pain, type of pain, location of infarct, impact of pain on daily life, and using pain-specific assessment tools along with multidomain assessment tools.

Our study has several strengths. Our dataset comprised more than 10,000 participants from 10 studies and took place across different stroke settings, thereby increasing overall generalisability of results when compared to studies based on single centres or settings. Similar work has established the moderate relationship between the EQ-5D pain domain and the Brief Pain Inventory, but was based on a much smaller sample size in a different health condition (40). Our population included people with language impairment due to stroke, thereby providing information on populations in whom measurement of pain can be particularly challenging. This population may be excluded from some types of clinical research due to challenges with consenting and following up these participants (43). Their inclusion improves the generalisability of our results. Relevant to our findings, both the EQ-5D 3 L and 5 L have been adapted for use in people with language impairment (44) and are available across a range of languages (45), should investigators choose to describe pain in their populations using this tool. Thus, in studies where pain is a potentially important outcome, or could be influenced by the intervention, researchers can report the pain item from the EQ-5D.

## Conclusion

Our findings demonstrate that even though relatively few studies included a pain-specific assessment, pain was still (inadvertently) captured moderately-well with a commonly used and accepted multidomain assessment tool (EQ-5D-3L). This may have implications when deciding on core outcome sets (COS) for stroke; a multidomain assessment tool such as EQ-5D that includes pain may be useful for inclusion in such a COS and has been recommended for inclusion in future sensorimotor recovery trials in stroke (46) and in the context of acute respiratory failure (47). However, caution should be applied as use of the EQ-5D has been validated in stroke populations with limited cognitive impairment and those without severe aphasia (48). Nevertheless, in the absence of consensus on pain measurement in stroke populations (1, 2), the widespread use and international applicability of the EQ-5D could lend itself to use in future studies where it is of interest to describe pain, but where pain is not the primary goal of the study. The EQ-5D pain domain should not replace more detailed pain assessment but may permit post-stroke pain to be described where resources and assessment burden hinder the implementation of additional, pain-specific assessments (40).

## Data availability statement

The data analysed in this study were obtained from the Virtual International Stroke Trials Archive (VISTA; https://www.virtualtrialsarchives.org/vista-acute/), the following licences/restrictions apply: access to these datasets is subject to approval by VISTA following completion of a Data Request Form. Requests to access these datasets should be directed to VISTA, vista.coordinator@glasgow.ac.uk.

## Ethics statement

The retrospective analysis of fully anonymised data was approved by the University of Glasgow’s Medical, Veterinary & Life Sciences College Ethics Committee (Project number 200170016). The original studies from which data were derived were conducted in accordance with the local legislation and institutional requirements. Written informed consent for participation in the current analysis was not required from the participants or the participants’ legal guardians/next of kin, as data were fully anonymised and extracted from a database of completed clinical trials.

## VISTA Steering Committees

Acute steering committee: K.R. Lees (Chair), A. Alexandrov, P.M. Bath, E. Bluhmki, N. Bornstein, C. Chen, L. Claesson, J. Curram, S.M. Davis, H-C. Diener, G. Donnan, M. Fisher, M. Ginsberg, B. Gregson, J. Grotta, W. Hacke, M.G. Hennerici, M. Hommel, M. Kaste (Emeritus), P. Lyden, J. Marler, K. Muir, C. Roffe, R. Sacco, A. Shuaib, P. Teal, N. Venketasubramanian, N.G. Wahlgren, and S. Warach. Rehab steering committee: M.C. Brady (Chair), M. Ali, A. Ashburn, D. Barer, A. Barzel, J. Bernhardt, A. Bowen, A. Drummond, J. Edmans, C. English, J. Gladman (Emeritus), E. Godecke, S. Hiekkala, T. Hoffman, L. Kalra, S. Kuys, P. Langhorne, A.C. Laska, K.R. Lees, P. Logan, B. Machner, G. Mead, J. Morris, A. Pandyan, A. Pollock, V. Pomeroy, H. Rodgers, C. Sackley, L. Shaw, D.J. Stott, K.S. Sunnerhagen, S. Tyson, P. van Vliet, M. Walker, and W. Whiteley. ICH steering committee: D.F. Hanley (Chair), K. Butcher, S. Davis, B. Gregson, K.R. Lees, P. Lyden, S. Mayer, K. Muir, and T. Steiner.

## Author contributions

MA: Data curation, Formal Analysis, Funding acquisition, Investigation, Project administration, Software, Writing – original draft, Writing – review & editing. HT: Formal Analysis, Investigation, Methodology, Writing – original draft, Writing – review & editing. MB: Conceptualization, Methodology, Writing – review & editing. TQ: Conceptualization, Data curation, Methodology, Writing – review & editing. KS: Data curation, Formal Analysis, Investigation, Writing – review & editing. NV: Data curation, Investigation, Writing – review & editing. AS: Data curation, Investigation, Writing – review & editing. AP: Data curation, Investigation, Writing – review & editing. GM: Conceptualization, Data curation, Funding acquisition, Writing – review & editing.
